# Metabolomic therapy response prediction in pretherapeutic tissue biopsies for trastuzumab in patients with HER2‐positive advanced gastric cancer

**DOI:** 10.1002/ctm2.547

**Published:** 2021-09-26

**Authors:** Thomas Kunzke, Fabian T. Hölzl, Verena M. Prade, Achim Buck, Katharina Huber, Annette Feuchtinger, Karolin Ebert, Gwen Zwingenberger, Robert Geffers, Stefanie M. Hauck, Ivonne Haffner, Birgit Luber, Florian Lordick, Axel Walch

**Affiliations:** ^1^ Research Unit Analytical Pathology Helmholtz Zentrum München–German Research Center for Environmental Health Neuherberg Germany; ^2^ Technische Universität München, Fakultät für Medizin, Klinikum rechts der Isar Institut für Allgemeine Pathologie und Pathologische Anatomie München Germany; ^3^ Genome Analytics Group Helmholtz Center for Infection Research HZI Braunschweig Germany; ^4^ Research Unit Protein Science and Metabolomics and Proteomics Core Helmholtz Zentrum München–German Research Center for Environmental Health Neuherberg Germany; ^5^ University Cancer Center Leipzig (UCCL) Leipzig University Medical Center Leipzig Germany; ^6^ Department of Oncology, Gastroenterology, Hepatology, Pulmonology and Infectious Diseases Leipzig University Medical Center Leipzig Germany

Dear Editor,

This study suggests for the first time a metabolomic classifier comprising molecules of DNA metabolism that is superior to conventional human epidermal growth factor receptor 2 (HER2) testing for predicting treatment response in routinely preserved pretherapeutic gastric cancer tissue biopsies.

Trastuzumab, a recombinant humanized monoclonal antibody directed against HER2, is the only targeted agent approved for the first‐line treatment of patients with HER2‐overexpressing advanced gastric cancer.^1^ Of the patients with advanced gastric cancer, up to 20% exhibit HER2 amplification or overexpression. However, only a subgroup of patients benefits from the addition of trastuzumab to chemotherapy. The overall response rate of the combined therapy is below 50%, indicating a considerable proportion of HER2‐amplified cancers are resistant to HER2 inhibition.^2^ At present, neither HER2 immunohistochemistry (IHC)^1^ nor HER2 in situ hybridization^2^ profoundly satisfies the prediction of trastuzumab therapy benefits in patients with advanced gastric cancer. Mass spectrometry imaging enables spatial metabolomics in routinely preserved gastric cancer tissue biopsies.^3^ We built a metabolomic classifier for HER2‐positive advanced gastric cancer applying spatial metabolomics and machine learning on routinely preserved pretherapeutic biopsies from a multicenter observational study.^4^ HER2 testing was centrally performed by IHC and in situ hybridization.^4^

The cohort (Table S1) was divided into therapy‐resistant and therapy‐sensitive patients by overall survival (Figure 1; cutoff = 13.8 months (ToGA^1^)). Two independent cohorts (Table S1) were used for the evaluation of the trastuzumab specificity of the metabolomic classifier. Patients with metastatic gastric cancer who underwent only chemotherapy (platin‐fluoropyrimidine), but not trastuzumab therapy, comprise one cohort (Table S1). The second cohort (Table S1) comprises the primary surgical resection specimens from patients with metastatic gastric cancer who neither receive chemotherapy nor trastuzumab therapy. Formalin‐fixed, paraffin‐embedded biopsies of all patients were analyzed by high mass resolution (Bruker Solarix 7.0T FT‐ICR MS; Bruker Daltonics) mass spectrometry imaging as previously described^3^ and processed using virtual microdissection of tumor cells. Random forest classifiers were trained using leave‐one‐out cross‐validation (Python 3.8, scikit‐learn 0.23.2). The training and prediction of the test set were repeated 100 times, and the majority vote was considered as the final prediction for each patient.

**FIGURE 1:**
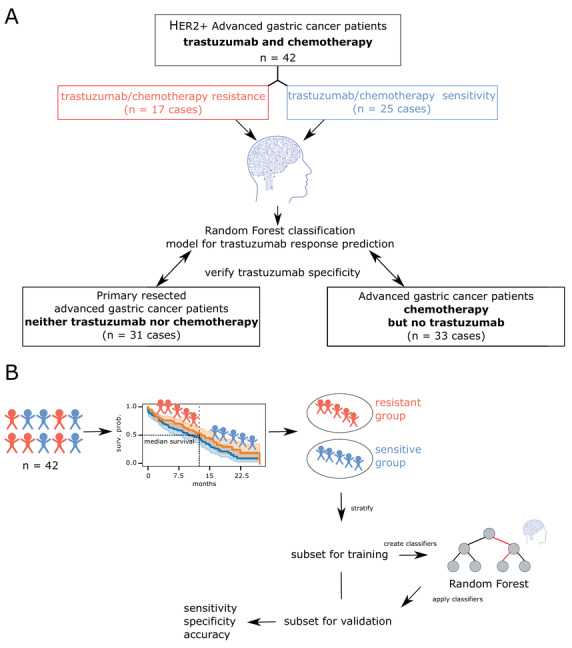
Study design. (A) A schematic representation of all included patient cohorts. Three independent patient cohorts were measured using MALDI mass spectrometry imaging. (B) All patients from the trastuzumab‐ and chemotherapy‐treated cohort were stratified into trastuzumab‐resistant and trastuzumab‐sensitive groups based on their survival data. The patient groups were further divided into training and validation sets. In the subsets, metabolites were used for the stratification between trastuzumab‐resistant and trastuzumab‐sensitive groups. The respective classifier was applied to the validation set, and sensitivity, specificity, and accuracy were calculated

Two metabolomic classifiers were created, one using only annotatable metabolites, a second allowing all metabolites. The first classifier reached an accuracy of 66.7%, while the second increased its accuracy to 73.8% for predicting trastuzumab response (Figure 2A). In contrast, HER2 IHC reached an accuracy of 57.1% for predicting trastuzumab response. The sensitivity for testing trastuzumab resistance by spatial metabolomics could be increased because the metabolome may take into account putative mechanisms of primary resistance to trastuzumab.

**FIGURE 2:**
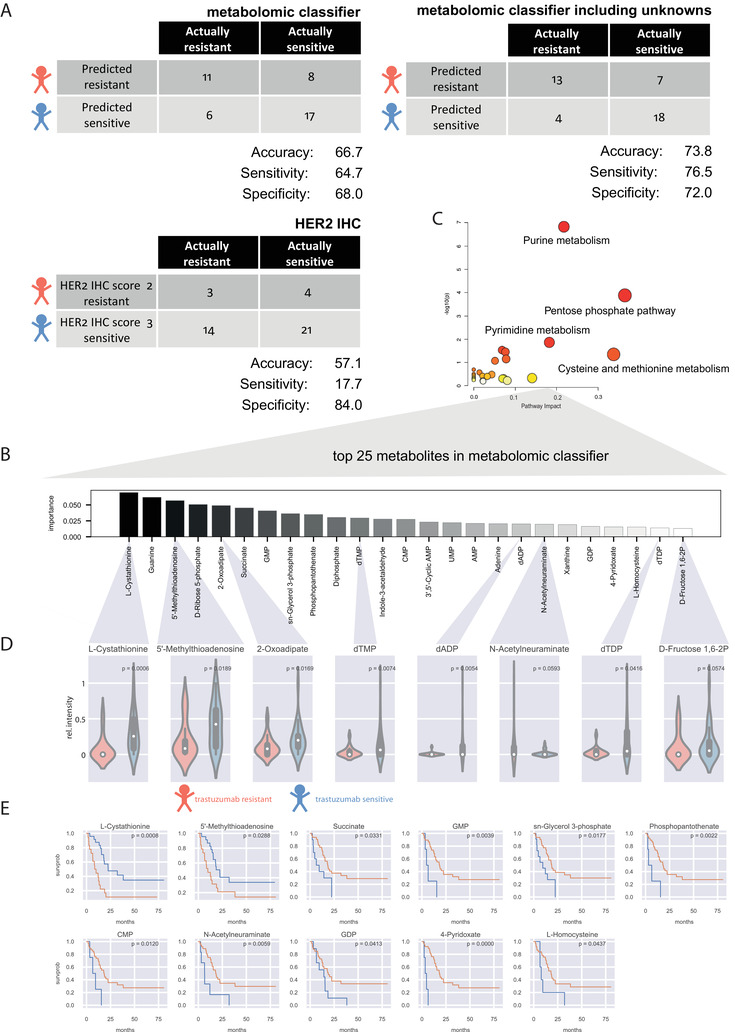
Performance of the metabolomic classifier, comparison with conventional HER2 testing, and evaluation of the most important metabolites in the classifier. (A) Confusion matrix visualization of prediction performance. Each row of the matrix represents the instances in a predicted class (trastuzumab‐resistant or trastuzumab‐sensitive), whereas each column represents the instances in an actual class. The sensitivity, specificity, and accuracy were calculated. (B) Importance plot of the 25 most important metabolites in the metabolomic classifier. (C) Pathway analysis including the 25 most important metabolites. (D) Evaluation for unequal distribution of the most important metabolites in trastuzumab‐sensitive and ‐resistant patients with advanced gastric cancer. *p*‐values were calculated using Mann‐Whitney‐*U*‐test. All significant metabolites were shown. (E) Evaluation for impact on patient survival of the most important metabolites in trastuzumab‐sensitive and ‐resistant patients with advanced gastric cancer. The blue line represents a high abundance of the individual metabolites, orange line low abundance. A log‐rank test was used for calculating *p*‐values. All significant metabolites were shown

Pathway enrichment analysis including the 25 most important metabolites (Figure 2B,C) indicated DNA metabolism as crucial to stratify patients into trastuzumab‐sensitive and trastuzumab‐resistant. Nucleotides revealed significantly higher quantities in trastuzumab‐sensitive patients (Figure 2D). In addition, higher levels of 5’‐methylthioadenosine were associated with good patient outcomes (Figure 2E). Amongst others, higher levels of GMP, CMP, and GDP were associated with poor patient outcomes. Our findings highlight that DNA metabolism showed an impact on response to trastuzumab. Nikolai *et al*. identified the anabolic metabolism of DNA as an important downstream effect of the HER2 oncogene in breast cancer.^5^ Consisting with this observation, we reveal for the first time that HER2‐driven change in DNA anabolism is important for HER2‐targeted trastuzumab therapy in patients with advanced gastric cancer. In addition, we investigated correlations within the most important metabolites in the classifier (Figure S2). The most important metabolite L‐cystathionine in the classifier also reveals associations to nucleotides, which might be due to its role in glutathione synthesis.

The impact of classifier metabolites on patient survival in independent cohorts was tested to evaluate the specificity of the metabolomic classifier to trastuzumab. Specific and significant prognostic effects in the trastuzumab‐treated cohort were revealed for GDP, CMP, 4‐pyridoxate, succinate, and 5’‐methylthioadenosine (Figure 3). Whether the predictive effect of biomarkers originates from chemotherapy or trastuzumab therapy remains unclear in previous studies. However, a strength of our study is the evaluation of the trastuzumab specificity of individual metabolites in the classifier. Abundances of all important metabolites in the treated patient groups can be obtained from Figure S3.

**FIGURE 3:**
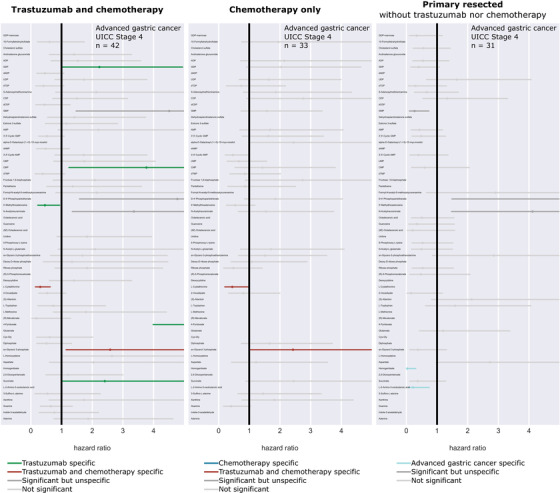
Validation of trastuzumab specificity by forest plot. The impact of all classifier metabolites on patient survival in three independent patient cohorts was tested to evaluate the specificity of the metabolomic classifier to trastuzumab. In addition to the presented human epidermal growth factor receptor 2 (HER2)‐positive trastuzumab‐ and chemotherapy‐treated patient cohort (cohort 1), patients who underwent chemotherapy only (cohort 2) and patients with primary resected advanced gastric cancer (cohort 3) were compared. Each row in the plot represents the same metabolite. The lines represent the univariate hazard ratios from Cox proportional hazards regression models with a 95% confidence interval for each metabolite in all individual cohorts. The hazard ratios based on an optimal intensity cutoff in the treated patient groups were shown. The metabolites GDP, CMP, 4‐pyridoxate, succinate, and 5’‐methylthioadenosine showed significant effects on patient survival exclusively in the trastuzumab‐treated cohort (green). Homogentisate and L‐2‐amino‐3‐oxobutanoic acid showed significant effects on outcome only in patients with primary resected tumors (blue). Moreover, two metabolites (L‐cystathionine and sn‐glycerol 3‐phosphate) reached significance in the trastuzumab‐ and chemotherapy‐treated cohort as well as in the chemotherapy‐only‐treated cohort (red)

For biological validation of DNA metabolism, NCI‐N87 (ATCC Cell Biology Collection) and MKN7 (Cell Bank RIKEN BioResource Center) were selected, as these cell lines are known as trastuzumab responder and non‐responder and mimic included patients.^6^ Proteins and RNA of the cell lines were analyzed as previously described.^7^, ^8^ The protein and RNA constitution also differed significantly between sensitive and resistant trastuzumab gastric cancer cells (Figure 4). Specifically, ribonucleoside‐diphosphate reductase large subunit (RRM1) was overexpressed in trastuzumab‐resistant cells, consistent with the DNA *de novo* pathway. In contrast, deoxycytidine kinase (DCK), a key enzyme for the DNA salvage nucleotide pathway, was significantly less expressed in trastuzumab‐resistant cells. In detail, the *de novo* synthesis and salvage pathway are the only possibilities in the cell to produce deoxyribonucleoside triphosphates, which are crucial for DNA synthesis.^9^ In the *de novo* synthesis, RRM1 plays a major role in maintaining the homeostasis of nucleotide pools.^9^ In the salvage pathway for producing deoxyribonucleoside triphosphates, the initial steps were catalyzed by TK1 and DCK.^10^ Taken together, our data suggest an alteration in both the *de novo* synthesis and salvage pathway that may be important for response to trastuzumab therapy.

**FIGURE 4:**
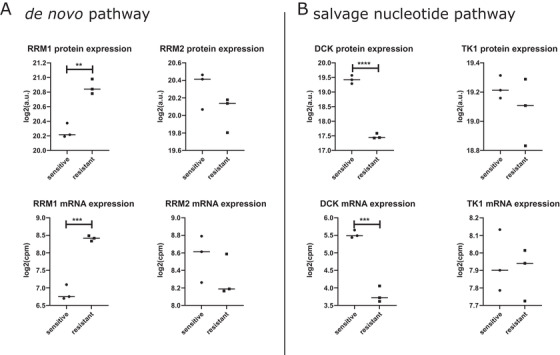
Protein and RNA analysis in relation to DNA metabolism in trastuzumab‐sensitive and ‐resistant gastric cancer cell lines. (A) Evaluation of key enzymes ribonucleoside‐diphosphate reductase large subunit (RRM1) and ribonucleotide reductase small subunit (RRM2) of the *de novo* DNA pathway in gastric cancer cells. RRM1 revealed a significantly higher abundance in the trastuzumab‐resistant gastric cancer cells (*p*_protein_ = 0.0018, *p*_mRNA_ = 0.0003). (B) Analysis for key enzymes deoxycytidine kinase (DCK) and thymidine kinase 1 (TK1) in the salvage nucleotide pathway. DCK was significantly increased in sensitive gastric cancer cells in comparison to resistant cells (*p*_protein_ < 0.0001, *p*_mRNA_ = 0.0003). a.u. = arbitrary units; cpm = counts per million

A previous approach, the AMNESIA study, uses genetic information to predict response to trastuzumab by analyzing gastric cancer tissue specimens.^11^ The predictive accuracy of the selected genes in the AMNESIA panel and HER2 IHC for predicting trastuzumab response was 76% and 65%, respectively. In our study, the accuracy of the metabolomic classifier and HER2 IHC for predicting trastuzumab response was 74% and 57%, respectively. In addition to the trastuzumab‐treated patients, our study includes two independent patient cohorts not treated by trastuzumab indicating trastuzumab specificity of metabolites constituting the classifier.

Metabolic information for the anti‐HER2‐therapy prediction can be also assessed by the non‐invasive ^18^F‐fluorodeoxyglucose (^18^F‐FDG)‐PET scan. Chen *et al*. performed ^18^F‐FDG‐PET/CT analysis on 64 patients with gastric cancer before surgical resection.^12^ Their results showed that the maximum standardized uptake value (SUV_max_) in gastric cancer was significantly lower when HER2 was expressed than when not expressed. The authors conclude that metabolic imaging has the potential to become a useful complement for assessing the molecular profile of gastric cancer and for predicting its response to anti‐HER2 antibody therapies, particularly in advanced gastric cancer with metastases, which may require neoadjuvant chemotherapy. Compared to spatial metabolomics, PET has the advantage of being non‐invasive. Obtaining biopsies for spatial metabolomics is an invasive method. However, it can be established on the already available routinely preserved pretherapeutic biopsies. Although PET/CT plays an important role in diagnostics, it is challenging to establish a cutoff for maximum standardized uptake values in the clinical setting. In contrast, the trastuzumab response prediction model by spatial metabolomics is based on decision trees of multiple parameters and metabolic pathways. Considering more parameters simultaneously increases the robustness of the prediction for trastuzumab response, which enhances the establishment in a clinical setting.

The current study illustrates a proof‐of‐principle because the patient number is limited for immediate clinical use. However, this study provides the first evidence that a tumor cell‐specific metabolomic classifier based on a clinical trial is superior for predicting trastuzumab response compared with conventional HER2 testing. Since trastuzumab is also well established in breast cancer, there is also potential for a metabolomic classifier for improved trastuzumab therapy response prediction.

## CONFLICT OF INTEREST

The authors have declared that they have no conflict of interest.

## AUTHOR CONTRIBUTIONS

T.K. and A.W. conceived the study design and wrote the manuscript. T.K. contributed to data acquisition, analysis, visualization, and interpretation. F.T.H., A.B., K.H. performed MALDI imaging preparations, measurements, and data analysis. K.E. and G.Z. performed cell experiments and interpretation. R.G. and S.M.H. performed RNA analysis, protein analysis, and interpretation. V.M.P. contributed to bioinformatics assistance. I.H. and F.L. contributed to patient characterization and to the provision of patient tissue and data. A.F., B.L., F.L., and A.W. supervised the project. All authors contributed to the review and approval of the manuscript.

## ETHICS APPROVAL

Approvals of the ethics committees of Leipzig University Medical Faculty and the ethics committee of the Technical University Munich were obtained.

## DATA AVAILABILITY STATEMENT

The datasets generated during and/or analyzed during the current study are available from the corresponding author on reasonable request.

## Supporting information

Supporting Information
