# Supplementary material for: Metabolomic therapy response prediction in pretherapeutic tissue biopsies for trastuzumab in patients with HER2‐positive advanced gastric cancer
Source: Clin Transl Med. 2021 Sep 26;11(9):e547. doi: 10.1002/ctm2.547 (PMC8473480; doi:10.1002/ctm2.547)
Supplement: Supplementary file 1 — Supporting Information [file CTM2-11-e547-s001.pdf]

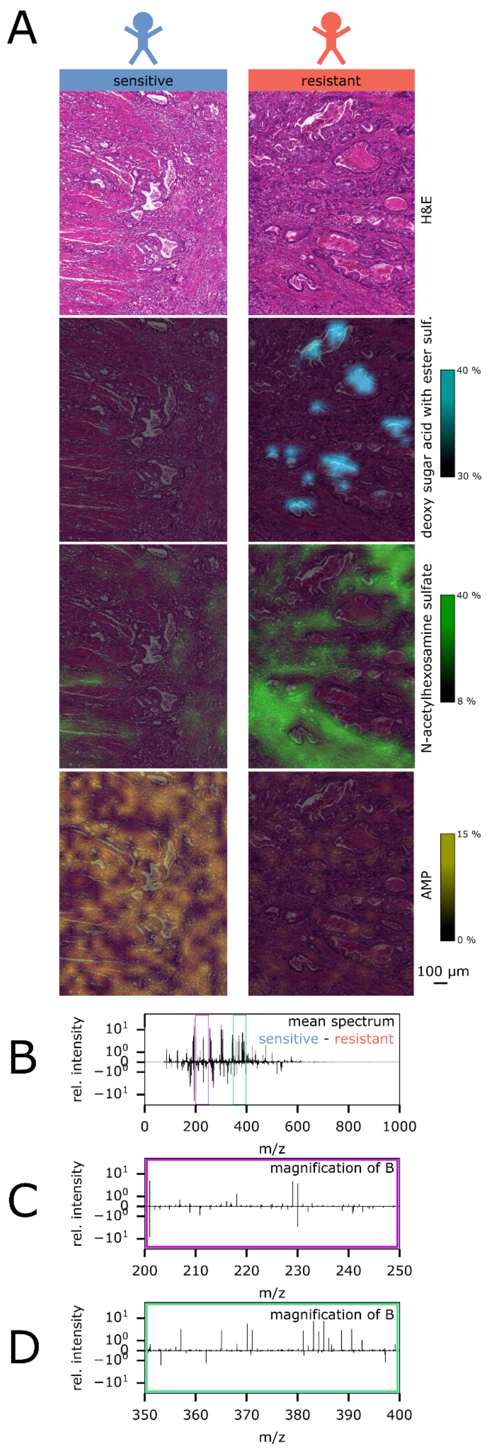

Supplemental Figure 1. Examples of changed metabolites between trastuzumab-resistant and trastuzumab-sensitive patient groups. A Ion map visualizations of deoxy sugar acid with ester sulfate (*blue*) and N-acetylhexosamine sulfate (*green*) revealed higher abundances in trastuzumab-resistant patients (*right column*). The quantities of both metabolites were lower in the tissues of trastuzumab-sensitive patients (*left*

column). On the contrary, AMP (*yellow*) revealed a higher abundance in tissues of sensitive patients compared with that of patients with resistant HER2-positive advanced gastric cancer. **B** Difference spectra of mean intensities between trastuzumab-resistant and trastuzumab-sensitive patient groups are exposing the 122 significantly changed metabolites in individual mass ranges. Zoom-in of the difference spectra **B** to the mass ranges **C** 200-250 *m/z* and **D** 350-400 *m/z*.

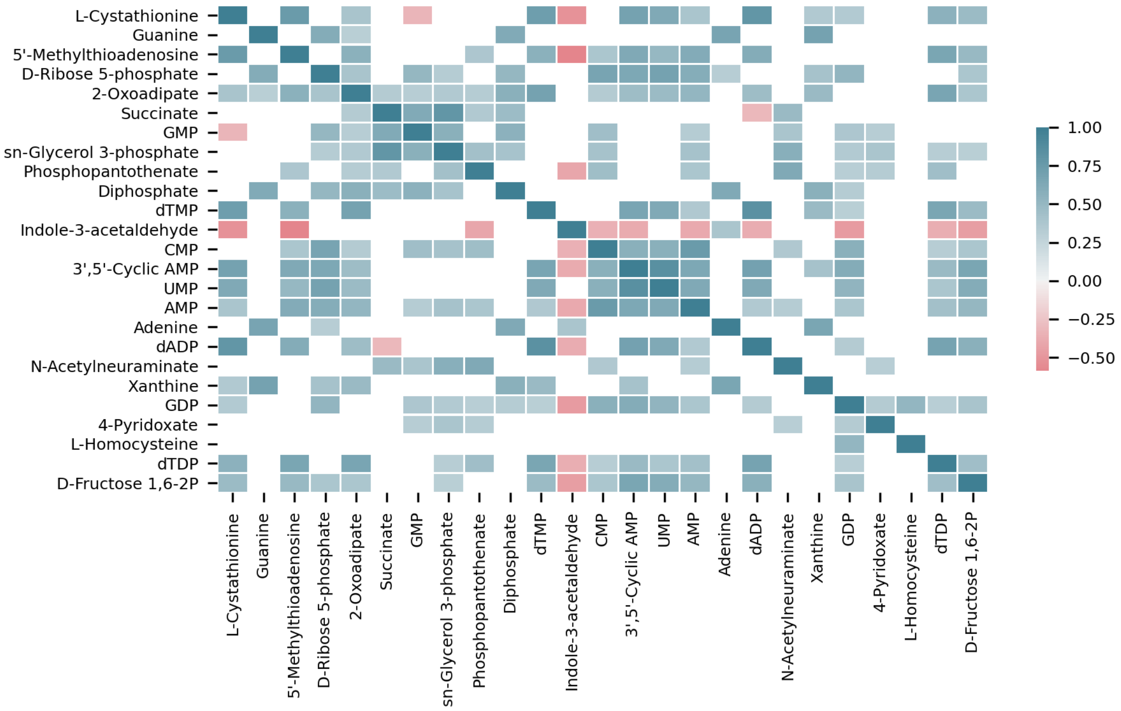

Supplemental Figure 2. Spearman's rank correlation for the 25 most important metabolites in the classifier. Blue squares are indicating significant positive correlation, red squares significant negative correlation. The color is associated with Spearman's rank correlation coefficient. Empty squares indicate that no significant correlation was found.

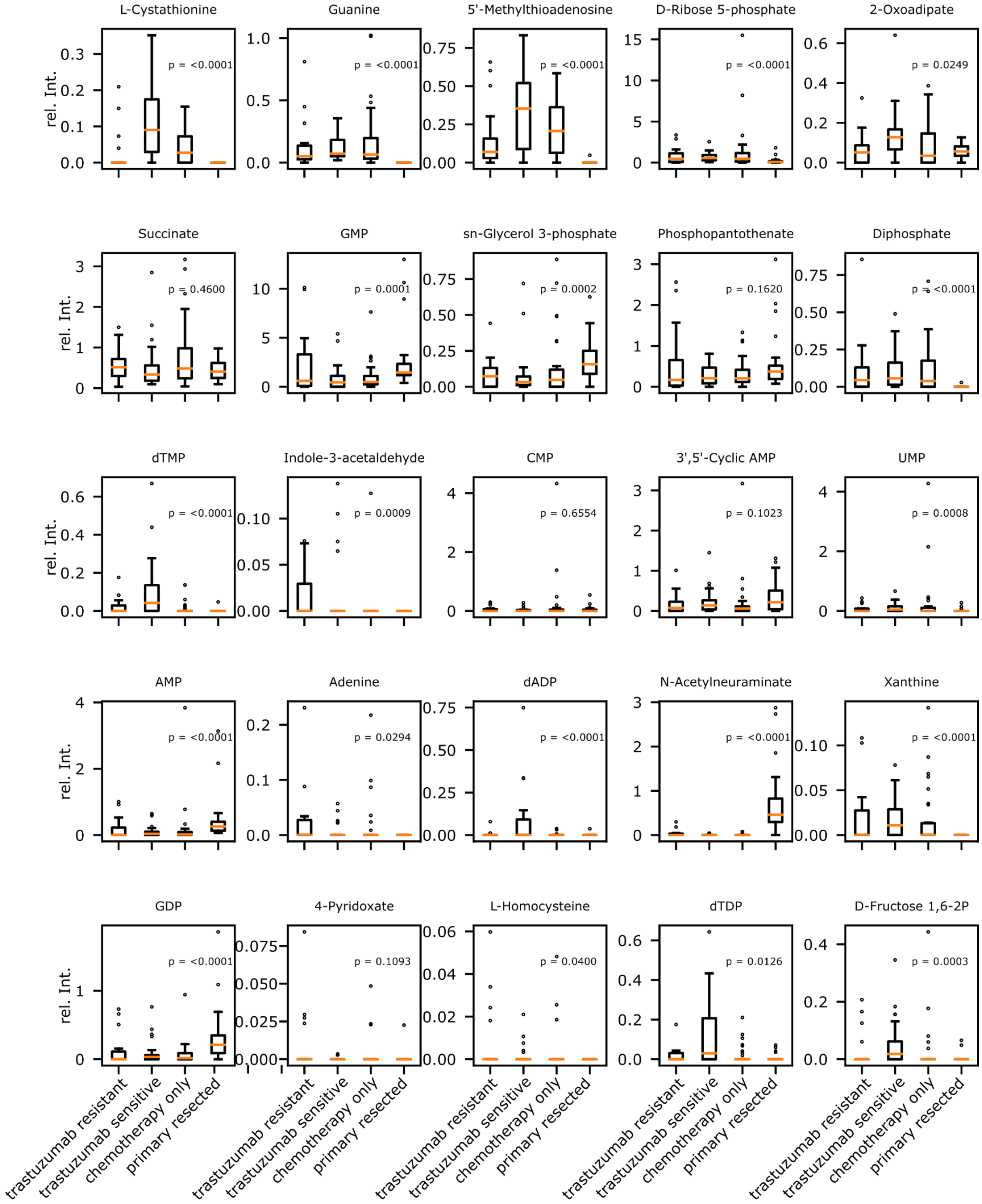

Supplemental Figure 3. Abundances of the 25 most important metabolites in the treated patient groups. P values are corresponding to the Kruskal–Wallis test per metabolite.

Supplementary Table 1. Baseline characteristics

		**Trastuzumab and chemotherapy**		**Only chemotherapy**	**No trastuzumab No chemotherapy**
		Resistant	Sensitive		
**Total**	106	17	25	33	31
**Gender**					
Male	79	12	21	24	22
Female	27	5	4	9	9
**Age, median (min-max)**	67 (24-86)	73 (24-86)	64 (46-79)	66 (37-85)	68 (34-82)
**UICC stage**					
IV	106	17	25	33	31
**Histotype**					
Intestinal type	47	6	13	9	19
Diffuse type	25	3	1	10	11
Mixed type	5	1	0	3	1
n.a.	29	7	11	11	0
**HER2 IHC score**					
0+	37	0	0	18	19
1+	14	0	0	7	7
2+	16	3	4	5	4
3+	39	14	21	3	1
***HER2* CISH**					
Amplified		16	24	3	n.a.
Non-amplified		0	0	28	n.a.
